# Hsp90 inhibitor induces autophagy and apoptosis in osteosarcoma cells

**DOI:** 10.3892/ijo.2014.2727

**Published:** 2014-10-23

**Authors:** MASAKI MORI, TOSHIAKI HITORA, OSAMU NAKAMURA, YOSHIKI YAMAGAMI, RYOSUKE HORIE, HIDEKI NISHIMURA, TETSUJI YAMAMOTO

**Affiliations:** Department of Orthopaedic Surgery, Kagawa University School of Medicine, Miki-cho, Kagawa 761-0793, Japan

**Keywords:** autophagy, apoptosis, Hsp90 inhibitor, osteosarcoma

## Abstract

Heat shock protein 90 (Hsp90) is constitutively expressed at 2-10-fold higher levels in tumor cells compared to normal cells, suggesting that it may be critically important for tumor cell growth and survival. These features make Hsp90 a potential target for anticancer drug development. Inhibition of Hsp90 activity not only results in rapid degradation of Hsp90 client proteins but also induces apoptosis of various tumor cells. Hsp90 also plays an important role in autophagy. An Hsp90 inhibitor induces autophagy through inhibition of mTOR. It is still under debate whether chemotherapy-induced autophagy in tumor cells is a protective response or is invoked to promote cell death. The aim of this study was to examine the effects of the Hsp90 inhibitor, geldanamycin (GA), on KTHOS osteosarcoma cells. We further examined whether a combination of GA and the autophagy inhibitor 3-methyl-adenine (3-MA) enhanced GA-induced apoptosis in KTHOS cells. GA had an inhibitory effect on cell proliferation and inhibited the Akt/mTOR signaling pathway in KTHOS cells. GA alone induced autophagy and apoptosis in KTHOS cells, but treatment with a combination of GA and 3-MA suppressed autophagy and induced apoptosis to a much greater extent than GA alone in these cells. It was considered that the autophagy inhibitor 3-MA suppressed a protective mechanism induced by Hsp90 inhibitor in tumor cells and induced apoptosis. Therefore, the combination of an Hsp90 inhibitor and an autophagy inhibitor may be an effective treatment for osteosarcoma because this combination effectively induces apoptotic pathways.

## Introduction

Osteosarcoma is the most common primary malignant bone tumor in children and adolescents, with 70–75% of cases occurring between the ages of 10 and 25 (1). Adult osteosarcoma is the second most frequent sarcoma with a low rate of response to current therapy due to inherent chemoresistance. The therapy of osteosarcoma has made good progress with the development of surgery and screening technologies and with the alliance of neoadjuvant chemotherapy, radiotherapy, immunotherapy and thermotherapy. However, problems of metastasis, recurrence and chemoresistance have not yet been solved. Indeed, metastases occur in >80% of individuals that are treated with surgery alone (2). Moreover, despite aggressive treatment including adjuvant and neoadjuvant chemotherapy, 30–40% of children die of osteosarcoma (3,4). There is therefore an urgent need to develop novel therapeutic agents.

Heat shock protein 90 (Hsp90) is an abundant molecular chaperone which constitutes 1–2% of total cellular protein. Hsp90 interacts with a variety of intracellular client proteins involved in cell growth, differentiation and survival, which facilitates their folding, activity, intracellular localization and proteolytic turnover (5,6). Hsp90 is abundantly expressed in eukaryotes and comprises >1% of the eukaryote total cellular content (7,8). However, Hsp90 is constitutively expressed at 2–10-fold higher levels in tumor cells compared to normal cells, suggesting that it may be critically important for tumor cell growth and survival (9). These features make Hsp90 a potential target for anticancer drug development. Geldanamycin (GA) is a benzoquinone ansamycin antibiotic that interferes with the action of Hsp90 leading to the degradation of Hsp90 client proteins. Since many of these client proteins are oncogenic proteins, GA inhibits the proliferation of cancer cells and shows anticancer activity in experimental animals. Inhibition of Hsp90 activity not only results in rapid degradation of Hsp90 client proteins but also induces apoptosis of various tumor cells (10,11). Currently several drug candidates that target Hsp90 are undergoing clinical trials for multiple indications, either as a single agent or in combination therapy (12). However, the molecular mechanism of Hsp90 inhibitors in cancer cells needs to be further elaborated.

Autophagy has recently gained attention because of its paradoxical roles in cell survival and cell death, particularly in the pathogenesis and treatment of cancer (13). Regulation of autophagy is highly complex with inputs from the cellular environment through the Akt/mTOR and MAPK/Erk1/2 signaling pathways (14). Hsp90 plays an important role in autophagy (15). An Hsp90 inhibitor induces autophagy through inhibition of mTOR (16). Autophagy is activated during starvation to provide an alternative energy source through self-digestion. Autophagy thus serves as a temporary survival mechanism. Autophagy is also important in the induction of tumor cell death (17) and excessive autophagy triggers autophagic cell death in tumors (18,19). It is still under debate whether chemotherapy-induced autophagy in tumor cells is a protective response or is invoked to promote cell death (14). However, recent studies have indicated that autophagy can function as a protective mechanism in cells that are exposed to antitumor agents and that blocking autophagy can trigger the activation of apoptosis (20–22). Based on these findings, it has been suggested that inhibitors of autophagy, such as the commonly used inhibitor 3-methyladenine (3-MA), might be an effective treatment for osteosarcoma because they activate apoptosis. Recent studies demonstrate that 3-MA promotes chemotherapeutic drug-induced apoptosis in cancer cells. Reportedly, 3-MA inhibits the activity of PI3K and blocks the formation of pre-autophagosomes, autophagosomes and autophagic vacuoles (23).

The aim of this study was to examine the effects of the Hsp90 inhibitor GA, on osteosarcoma cells. We investigated whether GA modulated the phosphorylation of proteins in the Akt/mTOR signaling pathway and/or induced autophagy or apoptosis in osteosarcoma cells. We further examined whether a combination of GA and 3-MA enhanced GA-induced apoptosis in osteosarcoma cells.

## Materials and methods

### Chemical reagents

GA was purchased from StressMarq Biosciences, Inc. (Victoria, BC, Canada), dissolved in dimethylsulfoxide (DMSO), and stored at −20°C. The inhibitor of autophagy, 3-MA, was purchased from Sigma Chemical Co. (St. Louis, MO, USA), dissolved in 1 mg/ml dimethylformamide (DMF), and stored at room temperature.

### Cell lines and cell culture

The KTHOS osteosarcoma cell line was used in this study. This cell line was grown in culture medium consisting of DMEM (Sigma-Aldrich, St. Louis, MO, USA) supplemented with 10% fetal bovine serum (FBS) (Sigma-Aldrich) and 100 U/ml penicillin. The cell line was routinely maintained at 37°C in a humidified 5% CO_2_ atmosphere. Cells were divided into four treatment groups; control (no inhibitor), GA, 3-MA and GA plus 3-MA (GA + 3-MA), for cell proliferation and the autophagy and apoptosis assays. Cells were seeded onto culture dishes and were cultured in growth medium for 48 h. The growth medium was then replaced with fresh medium with or without inhibitors. In the experiments that tested the combined effect of GA and 3-MA, cells were pre-treated with 10 mM 3-MA for 1 h before GA was added to the culture medium, and then cells were treated with 5 μM GA with 10 mM 3-MA for 24 h. In experiments testing the effect of GA or 3-MA alone, cells were treated with 5 μM GA or 10 mM 3-MA for 24 h.

### In vitro cell proliferation assay

Cell proliferation was determined using the CellTiter 96^®^ AQueous One Solution Cell Proliferation Assay (Promega Corporation, Madison, WI, USA). Cells were trypsinized and seeded at a density of 1×10^4^ cells/well in 96-well cell culture plates with 200 μl culture medium containing 10% FBS and were incubated for 48 h. Following this initial incubation, the growth medium was replaced with medium containing 10% FBS and GA at a concentration of 0, 0.01, 0.1, 1 or 10 μM. After 24 or 48 h, the medium was removed, the cells were washed with phosphate-buffered saline (PBS), and fresh medium containing the 3-(4,5-dimethylthiazol-2-yl)-5-(3-carboxymethoxyphenyl)-2-(4-sulfophenyl)-2H-tetrazolium (MTS) reagent (100 μl medium plus 20 μl MTS reagent/well) was added to each well. In the experiments that tested the combined effect of GA and 3-MA, cells were pre-treated with 10 mM 3-MA for 1 h before GA was added to the culture medium, and then cells were treated with 5 μM GA with 10 mM 3-MA for 24 h. In experiments testing the effect of GA or 3-MA, cells were treated with 5 μM GA or 10 mM 3-MA for 24 h. Cells in the four treatment groups with/without GA with/without 3-MA were also assayed for cell proliferation using this MTS assay. The optical density was measured at 490 nm using an automatic microplate reader after 2 h of further incubation following the addition of the MTS reagent. Absorbance is directly proportional to the number of living cells. Proliferation of each well was calculated as a percent of control. At least three independent experiments were performed for each assay.

### Western blot analyses

Cells were trypsinized and seeded at a density of 6×10^5^ cells/well in 6-well cell culture plates in 2 ml culture medium with 10% FBS. After 48 h, cells were treated with 10% FBS containing GA for the indicated time and at the indicated concentration. Cells of the four treatment groups were treated with/without GA with/without 3-MA. In the experiments testing the combined effect of GA and 3-MA, cells were pre-treated with 10 mM 3-MA for 1 h before GA was added to the culture medium, and then cells were treated with 5 μM GA with 10 mM 3-MA for 24 h. In experiments testing the effect of GA or 3-MA, cells were treated with 5 μM GA or 10 mM 3-MA for 24 h. Following treatment, the culture medium was replaced with lysis buffer (Cell Signaling Technology, Inc., Beverly, MA, USA), and cells were lysed on ice for 20 min. The cell lysates were spun at 15,000 × g using the Tomy MTX-150 centrifuge (Tomy Seiko Co., Ltd., Fukuoka, Japan) at 4°C for 30 min. The supernatant was then collected as the total cellular protein extract. Protein concentration was determined using the Protein Assay Bicinchoninate kit (Nacalai Tesque, Inc., Kyoto, Japan) and was standardized with bovine serum albumin. The samples of total cellular protein were loaded onto an SDS polyacrylamide gel (7.5, 10 or 12.5% commercial precast gel; Wako, Tokyo, Japan), and the proteins were separated by SDS-PAGE under reducing conditions. The mTOR, phospho-mTOR and cleaved PARP proteins were separated on a 7.5% SDS gel; the Akt, phospho-Akt, p70 ribosomal protein S6 kinase (p70S6K), phospho-p70S6K, p62/SQSTM1 and α-tubulin proteins were separated on 10% SDS gel; and 4E-binding protein 1 (4E-BP1), phospho-4E-BP1, microtubule-associated protein light-chain 3 (LC-3), cleaved caspase-9, and cleaved caspase-8 proteins were separated on a 12.5% SDS gel. The separated proteins were electrophoretically transferred to nitrocellose membranes (GE Healthcare Bio-Sciences Corp., Piscataway, NJ, USA). The membranes were blocked for 90 min in blocking buffer containing Tris-buffered saline-Tween-20 (TBS-T) and 10% EZ block (Atto Co., Ltd., Tokyo, Japan). The membranes were then incubated with primary antibodies, which were diluted in the blocking buffer, overnight at 4°C. Antibodies against Akt and phospho-Akt (Thr308) were purchased from Santa Cruz Biotechnology, Inc. (Santa Cruz, CA, USA). Anti-mTOR and anti-phospho-mTOR (Ser2448) antibodies were purchased from R&D Systems (Minneapolis, MN, USA). Antibodies against 4E-BP1, phospho-4E-BP1 (Ser65), p70S6K, phospho-p70S6K (Thr421/Ser424), cleaved caspase-9 (Asp315), cleaved caspase-8 (Asp391) and cleaved PARP (Asp214) were purchased from Cell Signaling Technology, Inc., and anti-α-tubulin antibody was purchased from Sigma-Aldrich. Anti-LC-3 and anti-p62/SQSTM1 antibodies were purchased from MBL Co., Ltd. (Nagoya, Japan). These primary antibodies are listed and characterized in Table I. The specific HRP-conjugated secondary antibody incubations were performed overnight at 4°C with gentle agitation. Bound antibodies were detected using the ECL Plus Western Blotting Detection system (GE Healthcare Bio-Sciences Corp.) and LAS-1000 Plus Image Analyzer (Fujifilm Co., Tokyo, Japan). Specific signals were quantified by densitometric analysis (Image J software).

### Flow cytometric TUNEL assay

TUNEL assay was performed using the MEBstain Apoptosis TUNEL kit Direct (MBL Co., Ltd.) following the manufacturer’s instructions. Cells were seeded at a density of 6×10^5^ cells/well and were cultured for 48 h. Cells of the four treatment groups were then treated with/without GA with/without 3-MA. In the experiments testing the combined effect of GA and 3-MA, cells were pre-treated with 10 mM 3-MA for 1 h before GA was added to the culture medium, and then cells were treated with 5 μM GA with 10 mM 3-MA for 24 h. In experiments testing the effect of GA or 3-MA, cells were treated with 5 μM GA or 10 mM 3-MA for 24 h. Cells were then washed gently three times in PBS containing 0.2% BSA, fixed with 4% paraformaldehyde for 30 min at 4°C, and washed twice in PBS containing 0.2% BSA. Next, 200 μl of 70% ethanol were added to the sample, which was mixed gently and then incubated for 30 min at −20°C. The samples were then washed twice in PBS containing 0.2% BSA, 30 μl of TdT solution were added and the samples were incubated for 1 h at 37°C. Next, the samples were washed twice in PBS containing 0.2% BSA, suspended to a final volume of 500 μl of PBS containing 0.2% BSA, and analyzed using a flow cytometer (FC-500; Beckman Coulter, Inc., Brea, CA, USA). Data are representative of three independent experiments.

### Determination of apoptosis using Annexin V-FITC and PI stain analysis

Cells were trypsinized and seeded at a density of 6×10^5^ cells/well in 6-well cell culture plates in 2 ml culture medium with 10% FBS and were then cultured for 48 h. Cells of the four treatment groups were treated with/without GA with/without 3-MA. In the experiments testing the combined effect of GA and 3-MA, cells were pre-treated with 10 mM 3-MA for 1 h before GA was added to the culture medium, and then cells were treated with 5 μM GA with 10 mM 3-MA for 24 h. In experiments testing the effect of GA or 3-MA, cells were treated with 5 μM GA or 10 mM 3-MA for 24 h. Cells were then incubated for 15 min in a dark room with Annexin V-FITC and PI using the Annexin V-FLUOS Staining kit (Roche Applied Science, Penzberg, Germany) according to the manufacturer’s recommendations. Stained cells were observed under a fluorescence microscope (Keyence Co., Osaka, Japan) equipped with a filter system (DAPI-BP for Annexin V: excitation wavelength 377 nm and detection 447 nm, TRITC for PI: excitation wavelength 543 nm and detection 593 nm). To quantify Annexin V and PI incorporation, at least 100 cells from each treatment group were examined under fluorescence microscopy, and the percentage of Annexin V-positive or Annexin V-plus-PI positive cells were calculated. The 100 cells sampled were chosen randomly to avoid bias.

### Detection of autophagic vacuoles using monodansyl cadaverine

Autophagic vacuoles were detected using mono-dansylcadaverine (MDC) by incubating cells with MDC solution (1:1,000 in Cell-Based Assay Buffer, 50 μM) in PBS using the Autophagy/Cytotoxicity Dual Staining kit (Cayman Chemical Co., Ann Arbor, MI, USA). Cells were seeded at a density of 6×10^5^ cells/well in 6-well cell culture plates in 2 ml culture medium with 10% FBS and were then cultured for 48 h. In the experiments testing the effect of GA, cells were treated with 5 μM GA for 24 h. Cells were then incubated with MDC for 15 min and immediately analyzed under a fluorescence microscope (DMI4000 B; Leica Microsystems, Wetzlar, Germany) using a fluorescence microscope equipped with a filter system (excitation wavelength of 460–500 nm, emission wavelength of 512–542 nm). Bright-field and fluorescence images were merged.

### Statistical analysis

All data and results presented are representative of, or calculated from, at least three independent experiments. For the cell proliferation assay, TUNEL assay, and Annexin V-FITC/PI stain analysis, differences between treatment groups were determined using the GraphPad Prism 5 for Windows software package. The data collected in three independent experiments for each group are expressed as means ± standard deviation (SD) and were statistically analyzed using ANOVA with Fisher’s PLSD post hoc test. P<0.05 was regarded as statistically significant.

## Results

### GA inhibits the proliferation of KTHOS cells

**Figure 1:**
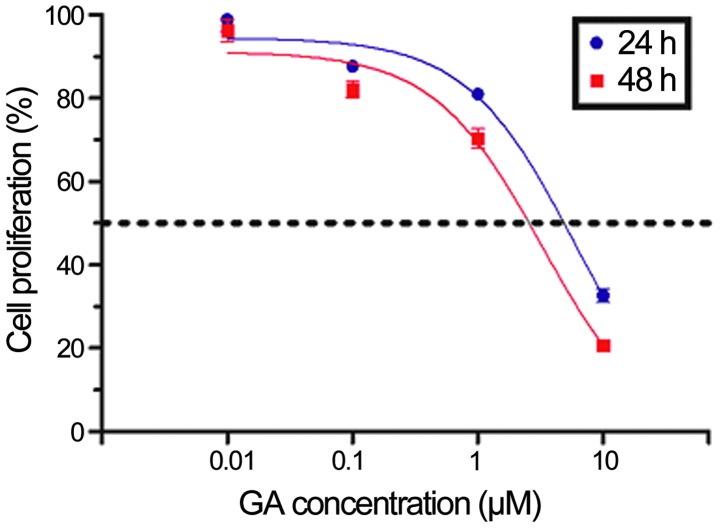
Geldanamycin (GA) inhibits the proliferation of KTHOS sarcoma cells. KTHOS cells were treated with various concentrations of GA for 24 or 48 h and cell proliferation was assessed using the 3-(4,5-dimethylthiazol-2-yl) -5-(3-carboxymethoxyphenyl)-2-(4-sulfophenyl)-2H-tetrazolium (MTS) assay. Proliferation is expressed as a percentage of control without GA.

Initially, we assessed the effects of GA on cellular proliferation using the CellTiter 96^®^ AQueous One Solution Cell Proliferation Assay. KTHOS cells were cultured in the presence of increasing doses of GA for 24 or 48 h. As shown in Fig. 1, GA inhibited KTHOS proliferation in a dose- and time-dependent manner. The IC_50_ value of GA at 24 h was 5.974 μM.

### GA induces autophagy in KTHOS cells by inhibiting Akt/mTOR/p70S6K signaling

**Figure 2:**
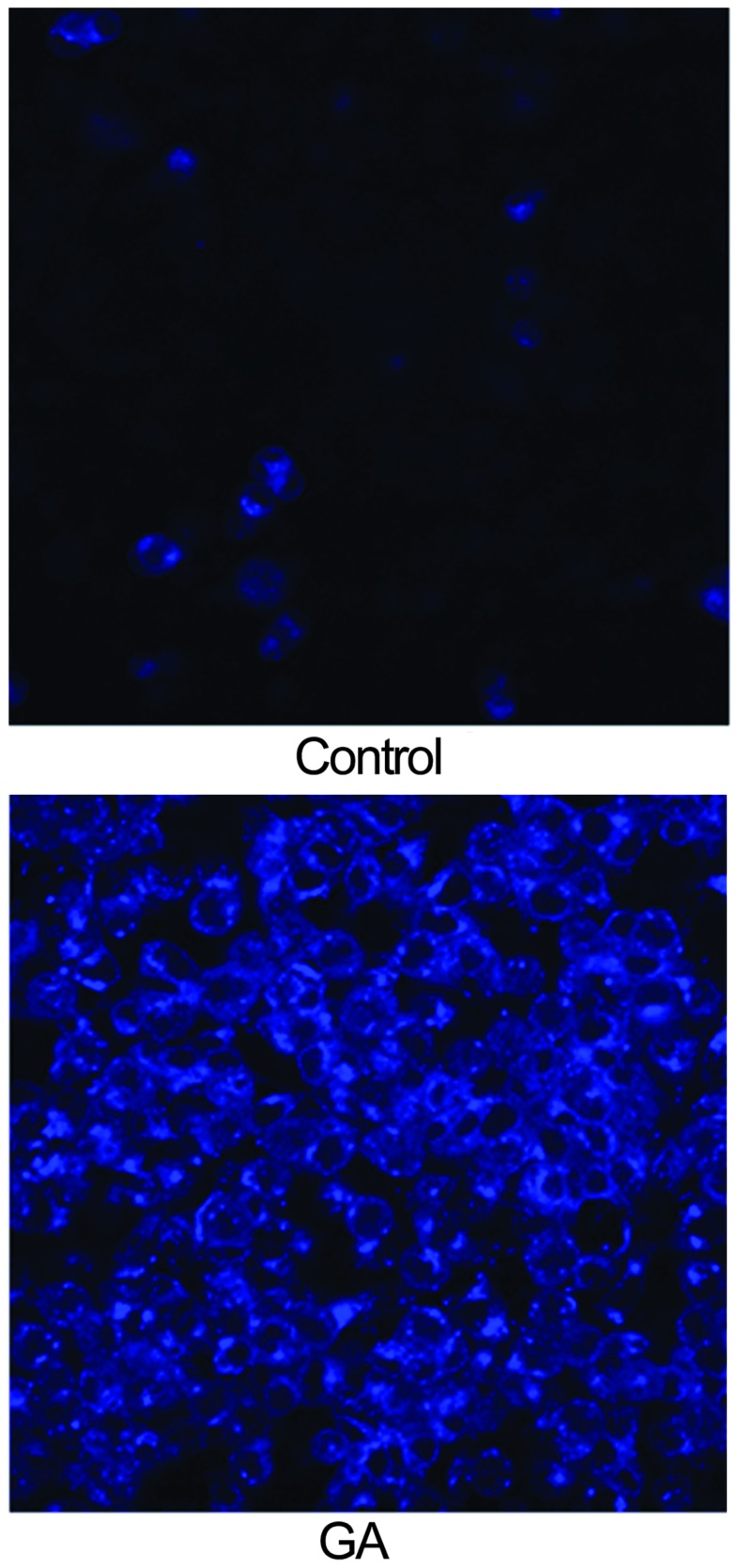
Geldanamycin (GA) induces autophagy in KTHOS cells. Autophagic vacuoles in KTHOS cells treated with or without 5 μM GA for 24 h were detected using monodansylcadaverine (MDC) staining.

**Figure 3:**
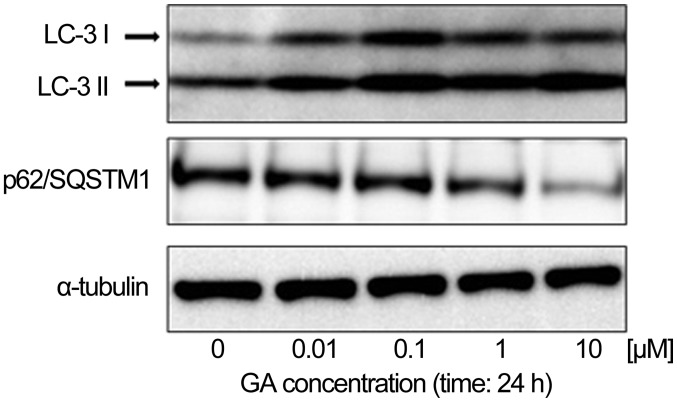
Geldanamycin (GA) induces autophagy in KTHOS cells. Western blot analysis of light-chain 3 (LC-3) and p62/SQSTM1 protein levels in KTHOS cells treated with various concentrations of GA for 24 h. α-tubulin was used as a loading control.

**Figure 4:**
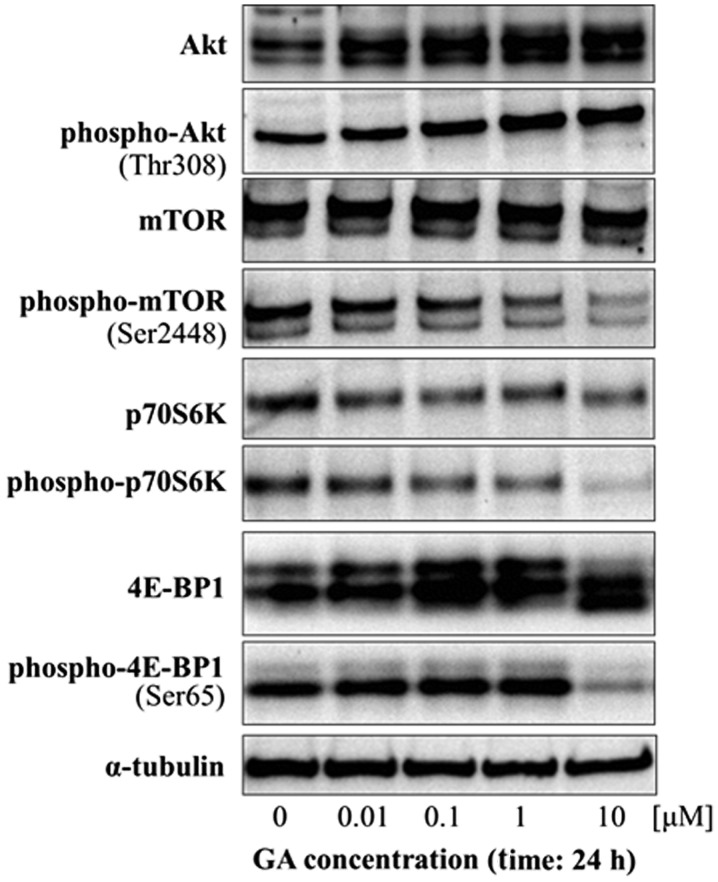
Geldanamycin (GA) inhibits the Akt/mTOR signaling pathway. Western blot analysis of Akt, phospho-Akt, mTOR, phospho-mTOR, p70 ribosomal protein S6 kinase (p70S6K), phospho-p70S6K, 4E-binding protein 1 (4E-BP1) and phospho-4E-BP1 in KTHOS cells treated with various concentrations of GA for 24 h. α-tubulin was used as a loading control.

We next investigated whether GA (5 μM for 24 h) induced autophagy in KTHOS cells. The concentration of GA chosen was based on the IC_50_ of GA after 24 h in the cell proliferation assay. For analysis of autophagy, we used MDC staining to detect autophagic vacuoles. MDC is an autofluorescent dye that accumulates in mature autophagic vacuoles, such as autophago-lysosomes, but not in the early endosome compartment; it is therefore a specific marker for autophagic vacuoles. Cells were incubated with MDC for 15 min after incubation with GA and were then analyzed using a fluorescence microscope. MDC fluorescence was observed in control and GA-treated KTHOS cells. However, GA-treated KTHOS cells displayed higher and more frequent accumulation of MDC accumulation than control cells (Fig. 2). These results suggest that GA treatment induces autophagy in KTHOS cells. To confirm that GA induced autophagy in these cells, we first used western blot analysis to analyze the expression of LC-3 and p62/SQSTM1 proteins, which are known to be upregulated and downregulated respectively in autophagy, in KTHOS cells exposed to concentrations of GA ranging from 0.01 to 10 μM, for 24 h. Fig. 3 shows that GA treatment induced a dose-dependent upregulation of LC3-II and downregulation of the p62/SQSTM1 protein, which confirms induction of autophagy in these cells. Activation of autophagy is associated with the Akt/mTOR/p70S6K signaling pathway in mammalian cells; Akt/mTOR/p70S6K signaling negatively regulates autophagy. We next examined the potential role of Akt/mTOR/p70S6K signaling in GA-induced autophagy by western blot analysis of the expression and phosphorylation of Akt and mTOR and of the downstream effectors of mTOR, p70S6K and 4E-BP1. GA did not cause any change in the levels of phospho-Akt in KTHOS cells. However, GA treatment resulted in a dose-dependent decrease in phospho-mTOR, phospho-p70S6K and phospho-4E-BP1 (Fig. 4). These findings indicate that GA affects the Akt/mTOR signaling pathway by inhibiting the phosphorylation of downstream effectors of mTOR.

The combined results indicate that GA induces autophagy by inhibition of the Akt/mTOR/p70S6K signaling pathway.

### GA induces the caspase-dependent apoptotic pathway in KTHOS cells

**Figure 5:**
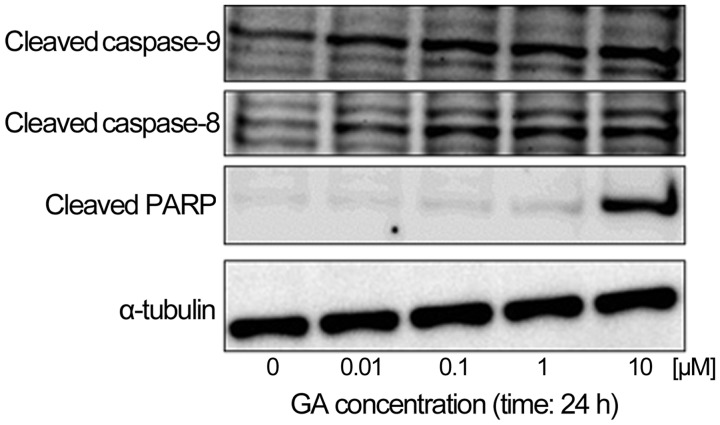
Geldanamycin (GA) induces apoptosis in KTHOS cells. Western blot analysis of caspase and PARP cleavage in KTHOS cells treated with various concentrations of GA for 24 h. α-tubulin was used as a loading control.

We next examined the effect of GA on caspase activity to determine if GA induces caspase-dependent apoptosis in KTHOS cells. Western blot analysis indicated that treatment of KTHOS cells with concentrations of GA ranging from 0.01 to 10 μM for 24 h resulted in dose-dependent cleavage of PARP, as well as activation of caspase-8 and -9 (Fig. 5). These results suggest the ability of GA to induce apoptosis in a caspase-dependent manner in KTHOS cells.

### GA potently inhibits the proliferation of KTHOS cells via induction of apoptosis following 3-MA pre-treatment

**Figure 6:**
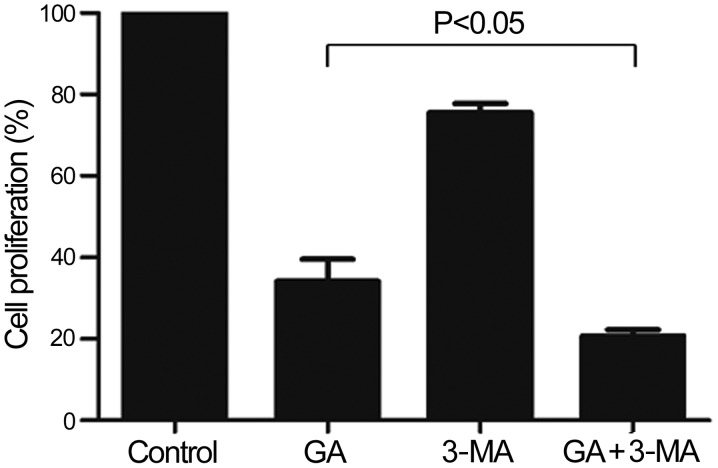
The combination of geldanamycin (GA) and 3-methyladenine (3-MA) potently inhibits the proliferation of KTHOS cells. KTHOS cells were pre-treated with or without 10 mM 3-MA for 1 h prior to treatment with or without 5 μM GA for 24 h and cell proliferation was assessed using the 3-(4,5-dimethylthiazol-2-yl)-5-(3-carboxymethoxyphenyl)-2-(4-sulfophenyl)-2H-tetrazolium (MTS) assay. Values are the mean ± standard deviation (SD) of three independent experiments.

**Figure 7:**
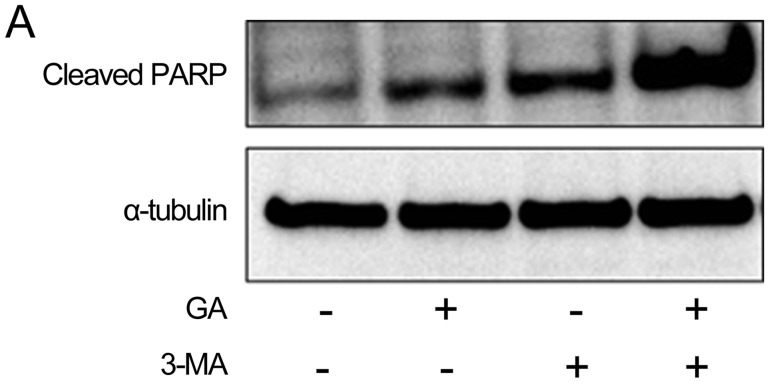

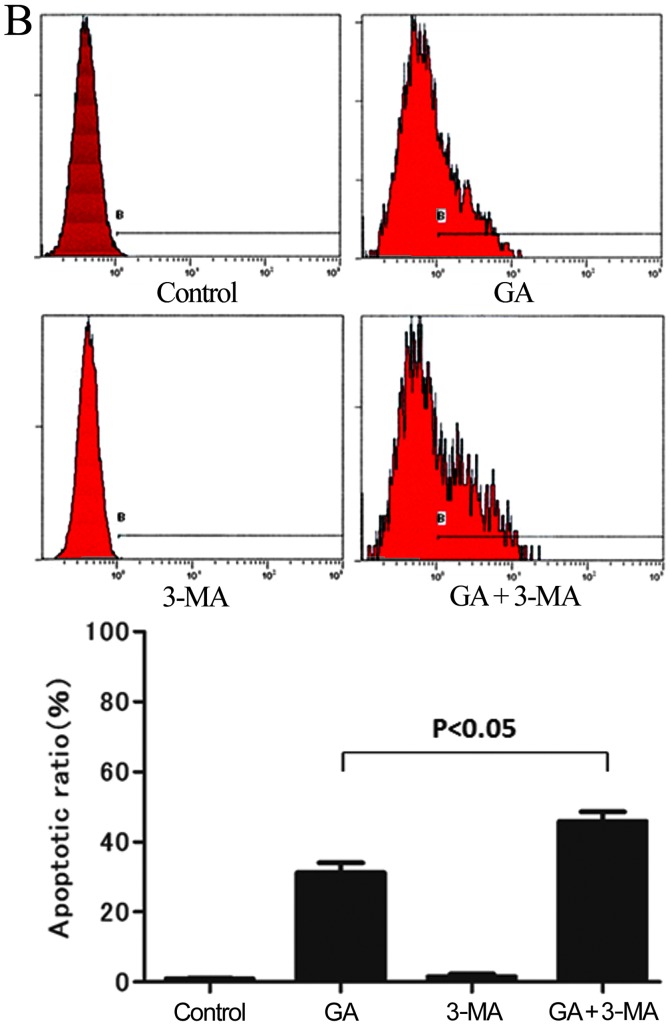

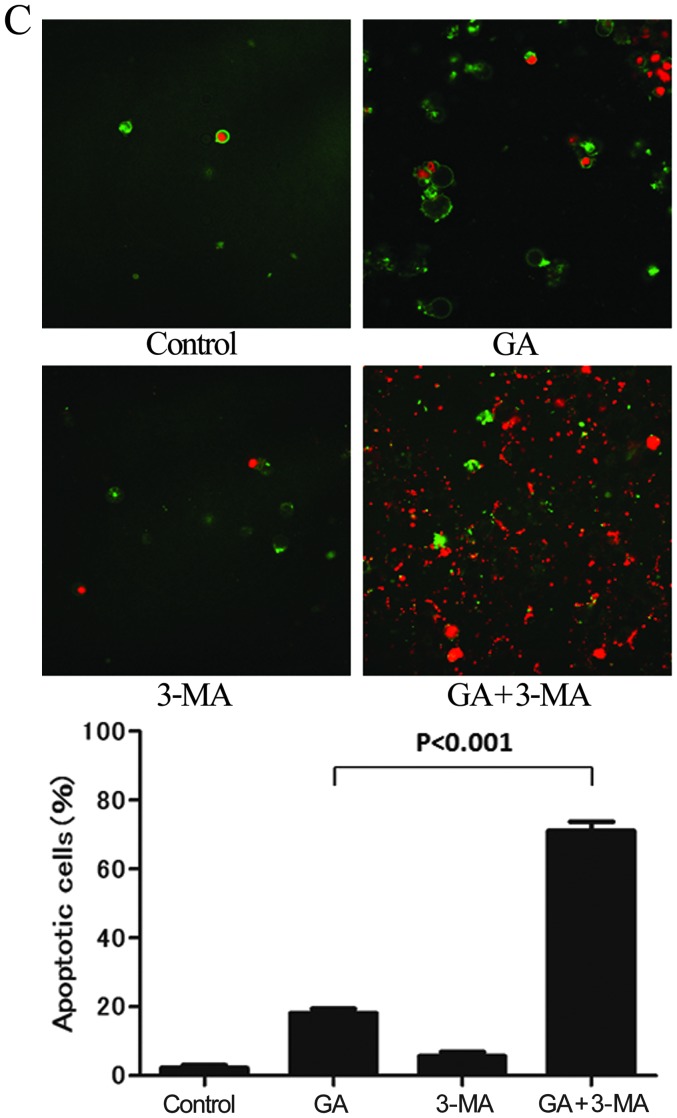
The combination of geldanamycin (GA) and 3-methyladenine (3-MA) potently induces apoptosis in KTHOS cells; (A) PARP assay. Western blot analysis of PARP cleavage in KTHOS cells pre-treated with or without 10 mM 3-MA for 1 h prior to treatment with or without 5 μM GA for 24 h. α-tubulin was used as a loading control. (B) TUNEL assay. The apoptotic rate of cells pre-treated with or without 10 mM 3-MA for 1 h prior to treatment with or without 5 μM GA for 24 h was quantified by flow cytometry using a TUNEL assay. Values are the mean ± standard deviation (SD) of three independent experiments. (C) Annexin V-FITC/PI staining assay. The percentage of cells pre-treated with or without 10 mM 3-MA for 1 h prior to treatment with or without 5 μM GA for 24 h was quantified using Annexin V-FITC/PI staining. Values are the mean ± SD of three independent experiments.

We next determined whether GA-induced autophagy is a protective or an apoptosis-promoting mechanism. For this purpose, we assessed cellular proliferation following pre-treatment of KTHOS cells with 10 mM 3-MA, which is commonly employed as a specific inhibitor of autophagic sequestration, for 1 h prior to administration of 5 μM GA for 24 h. As shown in Fig. 6, GA inhibition of KTHOS proliferation following 3-MA pre-treatment was significantly higher than that in the absence of 3-MA treatment (P<0.05). We next used western blot analysis to examine the effect of pre-treatment with 10 mM 3-MA 1 h on the effect of GA treatment (5 μM for 24 h) on protein expression of cleaved PARP, a marker of caspase-dependent apoptosis. As shown in Fig. 7A, pre-treatment of cells with 3-MA strongly increased the cleavage of PARP induced by GA. We next examined induction of apoptotic cells by GA and the effect of 3-MA pre-treatment on such induction. Apoptotic cells were assayed by flow cytometry using the TUNEL assay. The number of apoptotic cells induced by 24 h treatment with 5 μM GA was significantly increased by 10 mM 3-MA pre-treatment (P<0.05) (Fig. 7B). We also assayed apoptotic cells using Annexin V-FITC/PI staining and fluorescence microscopy. Annexin V is a marker of early apoptosis, and PI is a marker of late apoptosis and necrosis. As shown in Fig. 7C, the number of apoptotic cells as measured by this assay that were induced by 24 h treatment with 5 μM GA was significantly increased by 10 mM 3-MA pre-treatment (P<0.001). The combined results suggest that GA induces autophagy as a protective mechanism in KTHOS cells. Furthermore, GA potently inhibits the proliferation of KTHOS cells via induction of apoptosis following 3-MA pre-treatment.

## Discussion

Hsp90 is a molecular chaperone with several client proteins that are known to contribute to tumorigenesis. Hsp90 has recently been considered as a promising target for therapeutic intervention in a variety of cancers. The biological activity of GA and its semi-synthetic derivatives towards various hematopoietic neoplasms and solid carcinomas has been demonstrated *in vitro* and in murine xenograft models (24–27). Several clinical trials evaluating both GA derivatives and other novel Hsp90 inhibitors are ongoing. However, little is known regarding the potential activity of Hsp90 inhibitors in sarcomas. In this study, we demonstrate that GA inhibits the proliferation of human osteosarcoma KTHOS cells via induction of apoptosis and also induces autophagy. We further demonstrate that a combination of GA and 3-MA potently inhibits the proliferation of KTHOS cells to a greater extent than GA alone via induction of apoptosis.

We observed that GA induced time- and dose-dependent inhibition of proliferation of KTHOS cells. GA also induced apoptosis in KTHOS cells, resulting in altered cell morphology, DNA fragmentation, multiple caspase activation and PARP cleavage. Activation of caspase-8 indicated that the FasL/Fas pathway may be involved in GA-induced apoptosis. GA also activated caspase-9, which in turn, is known to activate the downstream effector caspase-3 and lead to PARP cleavage. The combined results suggest that GA-induced apoptosis is caspase-dependent.

Autophagy is a process in which subcellular membranes undergo dynamic morphological change (autophagosomes form and fuse with lysosomes) leading to the degradation of cellular proteins and cytoplasmic organelles. Autophagy plays a protective role when cells encounter environmental stresses such as starvation or pathogen infection (28,29). Autophagy also occurs under pathological conditions, such as in neurodegenerative disease or hereditary myopathies. Recent accumulating evidence indicates that autophagy often plays a role in malignant diseases. Specifically, autophagy is believed to play an important role in tumor development. During the early stages of tumor formation, autophagy functions as a tumor suppressor, and autophagic activity is often impaired in cancer cells. Many anticancer drugs which lead to apoptosis can also induce autophagy-related cell death in cancer cell lines (30,31). In the present study autophagy was demonstrated in GA-treated cells by MDC accumulation. GA treatment also induced dose-dependent upregulation of expression of the autophagy marker LC3-II. Inhibition of Hsp90 induces degradation of Hsp90 client proteins in cancer cells, and it is widely thought to lead to reduced proliferation. There are numerous Hsp90 client proteins. Akt is a known Hsp90 client protein. Akt is a serine threonine kinase that is downstream of PI3K and that has a large number of downstream targets implicated in survival and cell cycle regulation (32). In the present study, GA inhibited Akt/mTOR signaling, indicating that GA induces autophagy via targeting of Akt/mTOR signaling. The combined results suggest that GA-induced autophagy is associated with Akt protein degradation via a mechanism that is dependent on Hsp90 inhibition and on inhibition of Akt activation of mTOR.

3-MA is an inhibitor of autophagy. However, recent reports indicate that when 3-MA is combined with chemotherapeutic drugs it triggers apoptosis in some cancer cells (33). In the present study, we observed that the use of a combination of GA and 3-MA induced more cell death in KTHOS cells than the use of GA alone. We considered that autophagy can function as a protective mechanism in KTHOS cells that are subjected to GA and that blocking autophagy with 3-MA can promote the activation of apoptosis. It therefore appears that the combination of GA and 3-MA potently induced apoptotic cell death in KTHOS cells by inhibition of autophagy.

In conclusion, GA had an inhibitory effect on cell proliferation and inhibited the Akt/mTOR signaling pathway in KTHOS cells. GA also induced autophagy in KTHOS cells. However, treatment with a combination of GA and 3-MA suppressed autophagy and induced much higher apoptosis in KTHOS cells than GA alone. We considered that the autophagy inhibitor 3-MA suppressed a protective mechanism induced by Hsp90 inhibitor in the tumor cells and induced apoptosis. Therefore, the combination of an Hsp90 inhibitor and an autophagy inhibitor may be an effective treatment for osteosarcoma because this combination effectively induces apoptotic pathways.

## Figures and Tables

**Table I:** Primary antibodies used in western blot analysis.

Target	Source	Host	Dilution	Second antibody
LC-3	MBL Co., Ltd.	Rabbit	1:1,000	Anti-rabbit
p62/SQSTM1	MBL Co., Ltd.	Rabbit	1:1,000	Anti-rabbit
Akt	Santa Cruz Biotechnology, Inc.	Rabbit	1:1,000	Anti-rabbit
Phospho-Akt	Santa Cruz Biotechnology, Inc.	Rabbit	1:1,000	Anti-rabbit
mTOR	R&D Systems	Rabbit	1:1,000	Anti-rabbit
Phospho-mTOR	R&D Systems	Rabbit	1:1,000	Anti-rabbit
p70S6K	Cell Signaling Technology, Inc.	Rabbit	1:1,000	Anti-rabbit
Phospho-p70S6K	Cell Signaling Technology, Inc.	Rabbit	1:1,000	Anti-rabbit
4E-BP1	Cell Signaling Technology, Inc.	Rabbit	1:1,000	Anti-rabbit
Phospho-4E-BP1	Cell Signaling Technology, Inc.	Rabbit	1:1,000	Anti-rabbit
Cleaved caspase-9	Cell Signaling Technology, Inc.	Rabbit	1:1,000	Anti-rabbit
Cleaved caspase-8	Cell Signaling Technology, Inc.	Rabbit	1:1,000	Anti-rabbit
Cleaved PARP	Cell Signaling Technology, Inc.	Rabbit	1:1,000	Anti-rabbit
α-tubulin	Sigma-Aldrich	Mouse	1:1,000	Anti-mouse

LC-3, light-chain 3; p70S6K, p70 ribosomal protein S6 kinase; 4E-BP1, 4E-binding protein 1.
